# Up-Regulated Expression of Interferon-Gamma, Interleukin-6 and Tumor Necrosis Factor-Alpha in the Endolymphatic Sac of Meniere's Disease Suggesting the Local Inflammatory Response Underlies the Mechanism of This Disease

**DOI:** 10.3389/fneur.2022.781031

**Published:** 2022-02-23

**Authors:** Chao Huang, Qin Wang, Xueying Pan, Wei Li, Wei Liu, Wenqi Jiang, Li Huang, Anquan Peng, Zhiwen Zhang

**Affiliations:** ^1^Department of Otolaryngology-Head and Neck Surgery, The Second Xiangya Hospital, Central South University, Changsha, China; ^2^Department of Anesthesiology, Sun Yat-Sen University Cancer Center, Guangzhou, China

**Keywords:** Meniere's disease, endolymphatic sac, ES luminal fluid, cytokines, inflammatory

## Abstract

**Background:**

Immune mediated inflammatory changes affecting the endolymphatic sac (ES) may underlie the pathology of Meniere's disease (MD). The aim of the present study was to explore the differentially expressed cytokines in ES luminal fluid (ELF) of patients with MD, and the correlation between the expression of cytokines in the ELF with that in the serum was determined by quantitatively analyzing the cytokines in human ELF and serum.

**Methods:**

Human ELF, serum and ES tissues were collected from patients with unilateral MD and patients with acoustic neuroma (AN) during surgery. The Simoa Cytokine 6-Plex Panel kit was used to analyze the levels of cytokines in the ELF and blood samples of the patients. Immunohistochemistry and immunofluorescence were subsequently used to validate the relative expression levels of the cytokines in MD.

**Results:**

Significant differences were identified in the expression levels of interferon-γ (IFN-γ) (*P* < 0.001), interleukin (IL)-6 (*P* = 0.008) and tumor necrosis factor-α (TNF-α) (*P* = 0.036) in the luminal fluid of the ES comparing between the MD and AN groups. By contrast, the levels of IFN-γ, IL-10, IL-12p70, IL-17A, IL-6 and TNF-α in the serum of the MD group were not significantly different from those of either the AN group or healthy control subjects. In addition, no significant correlations in the expression levels of cytokines compared between the ELF and serum were found for the patients in either the MD or the AN group. Finally, the detection of positive expression of TNF-α, IL-6 and IFN-γ in the epithelial cells of the majority of ES specimens from patients with MD confirmed the up-regulated expression of these cytokines in the ES of patients with MD.

**Conclusions:**

The identification of up-regulated expression levels of TNF-α, IL-6 and IFN-γ in the ELF in the present study has provided direct evidence for an increased immunologic activity in the microenvironment of the ES in patients with unilateral MD, may suggest the local inflammatory response underlies the mechanism of this disease.

## Introduction

Meniere's disease (MD) is characterized by episodic vertigo, sensorineural hearing loss, and tinnitus or aural fullness. The primary pathology associated with MD is endolymphatic hydrops (EH) (1). Several of the clinical characteristics associated with MD suggest an underlying inflammatory or autoimmune etiology (2). While the endolymphatic sac (ES) has been convincingly shown to be involved in the initiation of inner ear immune responses (3, 4), various immunological factors have been shown to be involved with the ES, including the immunoglobulins IgG, IgM and IgA, as well as secretory components of the immune system found in the ES, and macrophages and plasma cells residing in the perisaccular connective tissue (2, 5). Whereas high-resolution immunohistochemistry shows that antigens reaching the ear may be trapped and processed by an immune cell machinery located in the ES, providing further proof of the central role of the ES in the immune function of the inner ear (6).

Previous studies have focused on the fact that the immune mediated inflammatory changes to the ES are associated with the production of EH in an animal model (7, 8), whereas the investigation of human ES in patients with MD revealed that mainly viral and autoimmune factors are involved in the pathological mechanism for MD (9–11). However, few studies have been published on the molecular pathomechanism(s) involved in the local alteration of the protein constituents and the composition of the luminal fluids of ES in patients with MD. As EH has been proposed to result from a disturbance in endolymphatic volume regulation that is associated with changes in the microenvironment of luminal fluid of the ES (12), understanding the molecular pathomechanism(s) underpinning the regulation of the immune reaction in the luminal fluids of ES is important both for furthering our knowledge of the physiopathology of EH and for identifying biomarkers for diagnosis or prognosis of pathologies of the MD. Recently, there has been a growing interest in developing a “liquid biopsy” of the inner ear as a surrogate for tissue biopsy to identify molecular biomarkers (13–16). Proteomics analysis in samples of perilymph (13) and ES luminal fluid (ELF) (14, 15) from patients with MD and controls suggested that increased inflammatory reactions in the inner ear may contribute to the pathology of MD. The results of the micro(mi)RNA expression profiles of perilymph from patients with MD and patients with otosclerosis indicated that 12 of the 16 miRNAs unique to MD were directly predicted to regulate inflammation and/or autoimmune pathways (16). However, The MD-specific changes accounting for the observed inflammation in the microenvironment of luminal fluid of the ES have to be validated through additional studies with a larger number of samples, also including the identification of the molecular target(s) of these samples from performing “liquid biopsy” in patients with MD.

**Figure 1:**
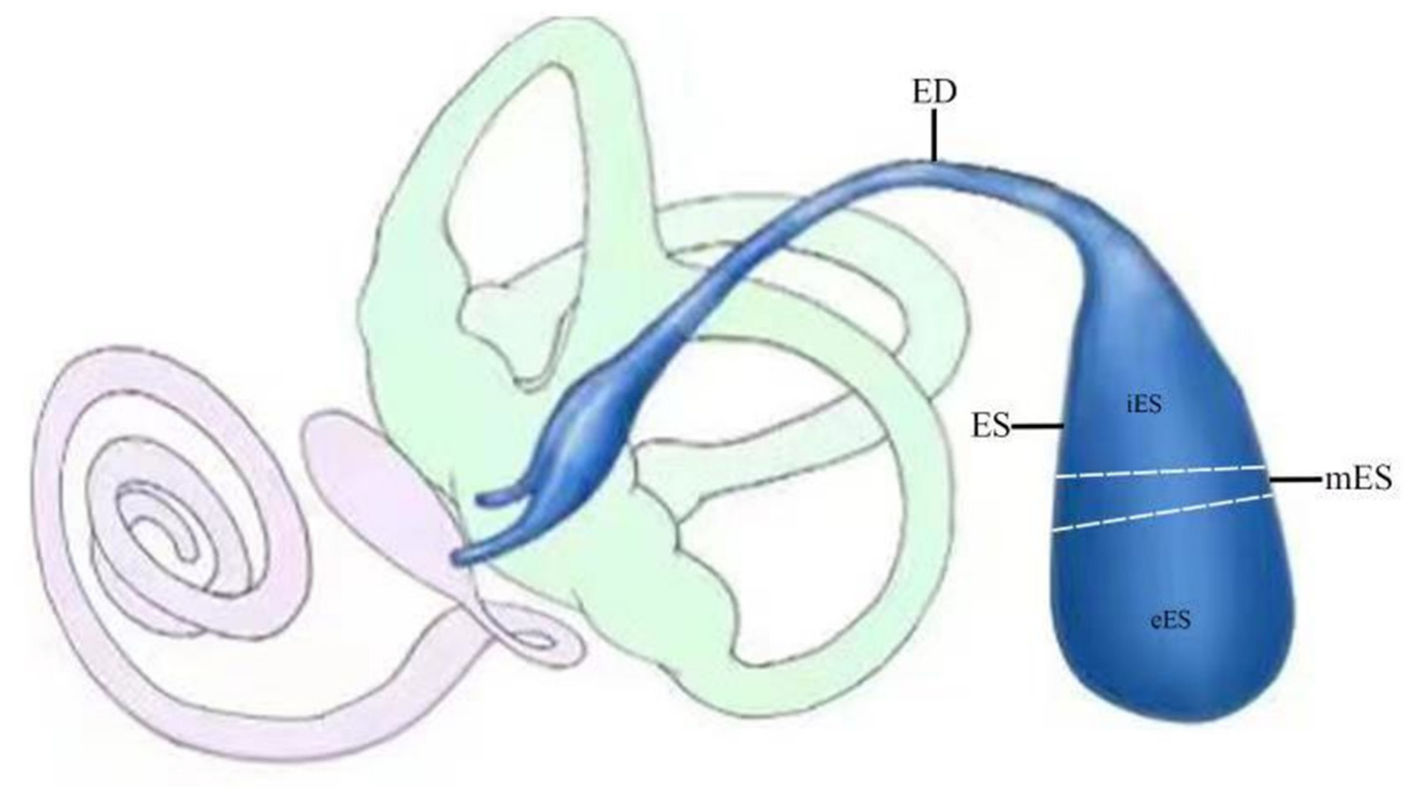
Schematic illustration of endolymphatic sac (ES; blue) and duct (ED; blue) and its relationship to the cochlea (pink) and the vestibule (light green). The sac can be divided into extraosseous (eES), intermediate (mES) and intraosseous (iES) portions.

The membranous labyrinth consists of the cochlea, vestibule and endolymphatic sac. The sac can be divided into extraosseous, intermediate and intraosseous portions, which is connected to the cochlea and vestibule by the endolymphatic duct (Figure 1). Theoretically, the use of cochlear and vestibular endolymph in addition to ELF should enable us to gain a fuller understanding of the processes involved; however, sampling the cochlear and vestibular endolymph in patients with MD would be impossible since the inner ear function of the patients would consequently be destroyed, and the protein concentrations in these compartments would be too low for the analysis (14). Therefore, sampling ELF has been the only viable approach for understanding the composition of inner ear fluid in patients with MD. However, since the amount of luminal fluid in the normal ES is so small (<4 μl) (17), obtaining enough endolymph in ES for analysis by direct aspiration has proven to be very difficult. Nevertheless, based on the fact that the protein concentration of luminal fluid in the ES is extremely high (~1,600 mg/dl), i.e., ~40-fold higher than that of cochleo vestibular endolymph (~38–60 mg/dl) as revealed in animal experiments (18), diluted luminal fluid from the ES would be suitable for use as a surrogate for endolymph in ES for screening the molecular targets of patients with MD. However, sampling luminal fluid of the ES from human subjects demands the highest levels of medical expertise and care to be taken in identifying the lumen of the ES, and in avoiding any contamination of the small sample of luminal fluid taken with the surrounding tissue fluids or with blood.

It has been previously reported that proinflammatory cytokines, such as tumor necrosis factor-α (TNF-α) and interleukin (IL)-1β, fulfill an important role in a plethora of middle and inner ear maladies (19, 20), including MD (21). However, the cellular contribution of these cytokines in the ES of MD is poorly understood, and it is not clear whether these cytokines are differentially expressed in the microenvironment of luminal fluid of the ES between patients with MD and control subjects. Additionally, it was reported that one third of MD cases may have a dysfunction of the immune system (22). Although clinical symptoms of systemic autoimmune disorder were not present in patients who were suspected to have autoimmune pathology-associated MD, studies have identified in these patients an excessive immune response due to an attack of the specific inner ear structures by cochlear innate immune cells that recognize self-antigens, which leads to the release of inflammatory cytokines, including TNF-α, IL-1β, interferon-γ (IFN-γ) and IL-17 (23–25). However, previous studies have shown that autoimmune inner ear disorder is confined mostly to the inner ear (23, 24), and whether there is an association between the systemic immune system and local inflammation in the microenvironment of luminal fluid of the ES in patients with MD remains unknown.

In the present study, the first direct quantification of the cytokine levels in the luminal fluid of the ES from patients with MD and patients with acoustic neuroma (AN), and in sera from patients with MD, patients with AN and healthy control patients, is provided in order to analyze the differentially expressed cytokines in the diluted luminal fluid of the ES between patients with MD and patients with AN. The correlation between cytokine levels of the diluted luminal fluid of the ES and of the sera of patients was also examined, and the findings were subsequently validated by analyzing the cellular distribution of cytokines that were expressed differentially in the ES of patients with MD using immunohistochemistry and immunofluorescence techniques.

## Patients and Methods

### Selection of Patients and Controls

In total, 20 patients diagnosed with unilateral MD according to the 2015 criteria of Classification Committee of the Bárány Society for the diagnosis of MD (26) were enrolled in the patient group. Samples of ES luminal fluid and peripheral blood were collected from 20 patients (8 males/12 females; mean age = 52.5 years, age range = 35–65 years) undergoing endolymphatic duct blockage (EDB) to treat their intractable MD with magnetic resonance imaging (MRI)-based visualization of unilateral EH at our University Hospital. In total, 20 patients with AN (11 males/9 females, mean age = 55.3 years, age range = 38–68 years) and 20 healthy volunteers (10 males/10 females, mean age = 48.3 years, age range = 28–62 years) were also enrolled as controls. ELF and peripheral blood were sampled from the 20 patients during the AN surgery via the trans-labyrinthine approach at our University Hospital, and peripheral blood was sampled from the 20 healthy controls. The controls with acoustic tumors had severe sensorineural hearing loss, but no history of sudden vertigo. The healthy volunteer controls had no history of sensorineural hearing loss or vertigo, and their audiograms showed normal hearing levels. None of the patients or healthy controls had a history of systemic autoimmune disease, and all of their laboratory parameters, including the electrocardiogram, chest radiography, blood cell counts (red blood cells, white blood cells and platelets), liver and kidney function tests and urinalysis, were normal. The gender distribution and mean ages of the patients and controls were not significantly different (*P* < 0.05 for the chi square test and t test).

### Sampling of ELF and Serum

**Figure 2:**
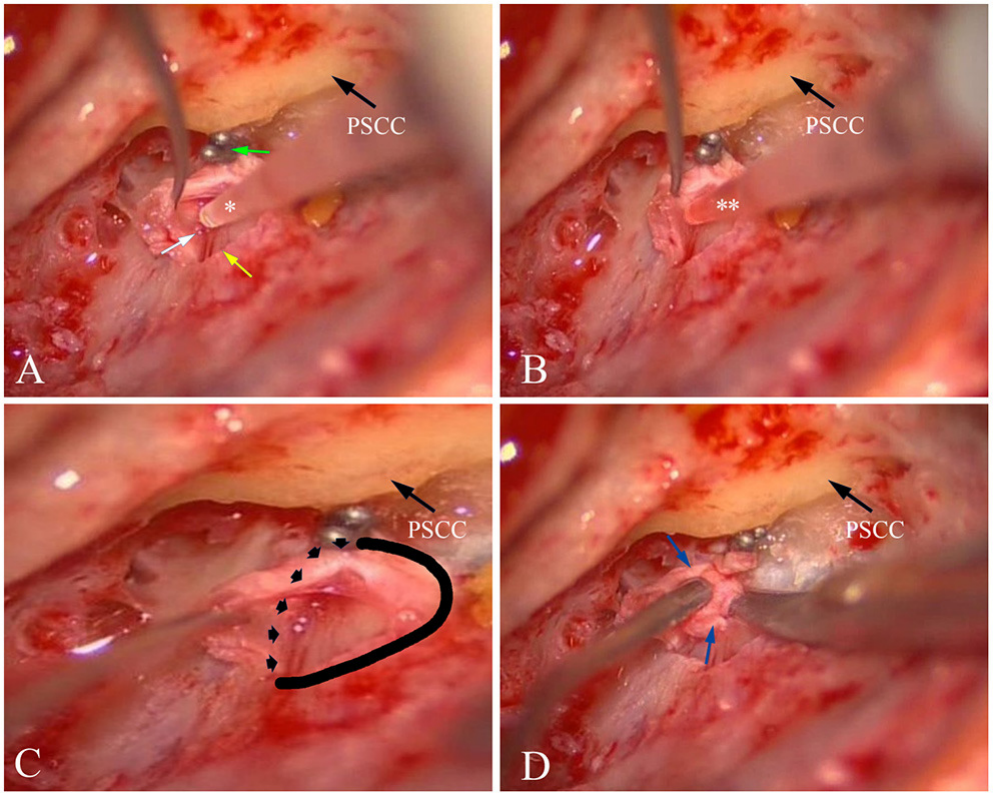
Sampling ELF and collecting ES specimen from patient no. 8 with left MD. **(A)** After blockage of the distal intraosseous portion of the ES using two titanium clips (green arrow) and exposure of the lumen of the ES (yellow arrow), a small amount of fluid (endolymph) could be found (white arrow). Then, 2 μl sterile water (*) was infused into the lumen of the ES. **(B)** 2 μl diluted luminal fluid (**) was aspirated from ES. **(C)** A solid and dotted line was used to mark the border of the ES specimen that would be resected. **(D)** An ES specimen including the distal intraosseous and proximal extraosseous (lateral side) portions (blue arrow) of the ES was resected. PSCC, posterior semicircular canal; ES,endolymphatic sac; ELF, ES luminal fluid; MD, Meniere's disease.

The EDB procedures used for treatment of MD, as described previously (27), were performed in 25 patients with intractable MD with MRI based visualization of unilateral EH between January 2019 and June 2020. After blockage of the distal intraosseous portion of the ES by applying the ligating clip, the proximal extraosseous ES was opened with an L-shaped incision, and the lumen of the ES was carefully identified (Figure 2A). As the amount of luminal fluid is very small (<4 μl) and the luminal fluid is distributed non-uniformly in the lumen of the sac, direct aspiration of luminal fluid proved to be very difficult. Consequently, 2 μl sterile water was infused into the lumen of the ES (Figure 2A), and then 2 μl diluted luminal fluid was aspirated from the lumen of the ES (Figure 2B) using a calibrated (1–5 μl) disposable micropipette (Eppendorf, Hamburg, Germany) with an inner diameter of 0.4 mm and an outer diameter of 0.8 mm at the tip, which had previously been scaled with a 2 μl marker and strictly sterilized. The sample was transferred into an Eppendorf tube that had been prefilled with 100 μl sterile water, immediately placed on ice and transferred into an −80°C freezer before further analysis. To avoid contamination by other body fluids, the surgical area was dried using suction and the careful application of haemostatics (13–15). The samples were macroscopically controlled for possible blood contamination, and were discarded if the samples were shown to possess even slightly red-colored fluid. A total of 20 samples of ELF from 25 patients with MD receiving EDB were obtained, as the lumen of the ES in 4 patients with MD could not be identified due to possible hypoplasia or fibrosis of the sac, and one sample was excluded due to possible blood contamination. A total of 20 control samples of ELF from 23 patients with AN were obtained during acoustic tumor surgery via a trans labyrinthine approach between January 2019 and June 2020, as the lumen of ES in one patient was poorly exposed for sampling, and two samples had to be abandoned due to the suspicion of blood contamination.

Blood was sampled from 20 patients with MD and 20 patients with AN, from whom the diluted luminal fluid of the ES was sampled, and 20 healthy volunteer controls for serum cytokine analysis. Following centrifugation at 10,000g for 20 min at room temperature, the supernatant was transferred into an Eppendorf tube and stored at −80°C.

### Immunoassay Based on Single Molecular Array

The Simoa Cytokine 6-Plex Panel 1 Advantage kit (cat no. 102958; Quanterix Corp. Billerica, MA, U.S.A.) was used to analyze the cytokines in the diluted luminal fluid of the ES and blood samples. This kit comprises a digital immunoassay based on single molecular array (Simoa) that quantifies the levels of the following human cytokines: IFN-γ, IL-10, IL-12p70, IL-17A, IL-6 and TNF-α. After thawing, the samples were vortex mixed and spun at 10,000 × g for 5 min; subsequently, the supernatant (50 μl) was directly transferred to a Quanterix supplied 96 well plate. The corresponding calibration curves for IFN-γ, IL-10, IL-12p70, IL-17A, IL-6 and TNF-α were constructed, and transferred to the 96 well plate. Assay ranges (pg/ml) were found to be 0.137-353 for IFN-γ, 0.09-152 for IL-10, 0.062-145 for IL-12p70, 0.034-55 for IL-17A, 0.278-500 for IL-6 and 0.176-150 for TNF-α. Control samples and the inter-assay control were also all transferred to the 96-well plate. The 96-well plate was loaded on board, and the desired dilution factor for the samples was created using the Simoa HD-1 Analyzer. Utilizing a 3-step procedure in a reaction cuvette, target antibody-coated paramagnetic beads were combined and incubated with sample alone. Target molecules present in the sample were captured by the antibody coated beads. After washing, biotinylated detector antibodies were mixed and incubated with the beads. The detector antibodies subsequently bound to the captured target during this incubation. Following a second wash, streptavidin-conjugated β-galactosidase (SBG) reagent was added, binding the biotinylated antibodies and leading to SBG enzyme-labeling of the captured cytokine proteins. Following a final wash, the beads were resuspended in resorufin β-D-galactopyranoside (RGP) reagent, transferred to a Simoa disk array and sealed. The cytokine proteins captured by the antibody-coated paramagnetic beads and labeled with the SBG reagent hydrolyzed the RGP substrate, yielding a fluorescence signal. The fluorescence signal values generated from the calibration curve of known concentrations were fitted using a 4-parameter logistic curve and 1/y^2^ weighting. Finally, the concentration of cytokines was determined from their fluorescence signals fitted to the calibration curve.

### Tissue Preparation and Immunohistochemistry

#### Tissue Preparation

i) Sac specimens from patients with MD. EDB surgery provided the opportunity to obtain the sac specimens. A total of 9 small pieces of the intermediate portion of the ES, including the distal intraosseous and proximal extraosseous (lateral side) portions of the ES tissue (Figures 2C,D), were obtained from patients undergoing EDB surgery between October 2019 and June 2020. The patients comprised 5 cases with stage III, and 4 cases with stage IV MD, according to staging on the pure tone average at 0.5–3 kHz (28).
ii) Sac specimens from patients with AN. Fresh tissue samples of the human ES were collected during surgery for AN using the trans-labyrinthine approach. After sampling ELF, the bone tissue surrounding ES was dissected and thinned using diamond drills of various sizes. Subsequently, both the intraosseous and proximal extraosseous portions of the ES tissue were resected following removal of a thin shell of bone around the ES, with the exception of the endolymphatic duct and the part of the ES located on the sigmoid sinus. A total of 10 ES specimens from 10 patients were collected between January 2019 and June 2020.

#### Immunohistochemistry Analysis

Subsequently, tissues were fixed with 4% paraformaldehyde solution for 12 h, and then transferred sequentially to 50, 70, 95 and 100% ethanol baths (1 h each bath) for dehydration. Then, samples were infiltrated by molten paraffin wax in the oven for 1 h, before being embedded into paraffin wax blocks. Paraffin-embedded ES tissues were sectioned at 5-μm thickness for subsequent immunohistochemistry. Slides were deparaffinized and rehydrated in 3% hydrogen peroxide for 5 min. After antigen retrieval by 1% sodium dodecyl sulfate in PBS for 10 min, slides were blocked in 10% horse serum (Abcam) in PBS for 1 h. Primary antibodies (100 μl, 1:400 dilution) against IL-6 [Cusabio Biotech Co., Ltd., TX, USA; cat. no. CSB PA06757A0Rb, rabbit anti-*Homo sapiens* (human) IL-6 polyclonal antibody], IL-10 [IL-10, Abcam, Cambridge, UK; cat. no. AB189392, Rat monoclonal (JES5-2A5)], IFN-γ [Thermo Fisher Scientific, Inc., Waltham, MA, USA; MM700B, murine recombinant] and TNF-α [Abcam, Cambridge, UK; cat. no. AB183218, rabbit monoclonal (EPR19147) to TNF-α] in 0.1% Triton-PBS (PBST) were applied to the samples overnight at room temperature. Negative control samples were processed simultaneously in an identical manner, with the exception that PBST was used to replace primary antibody. Slides were incubated with horseradish peroxidase-conjugated secondary antibody (Beyotime Institute of Biotechnology, Jiangsu, China; 1:200) for 1 h, and then washed three times in PBS. After gently washing in PBS three times, the samples were incubated in diaminobenzidine solution (FUJIFILM Wako Pure Chemical Corporation, Osaka, Japan; 1:50) for visualization. Finally, after washing with PBS, the samples were dehydrated and mounted with cover glasses. The immunohistochemical assessment was performed and agreed upon by two pathologists who were blinded to all patient clinical data. The positive immunoreactivity localized at the plasma membrane of epithelial cells was evaluated. Staining was scored for intensity (0: no staining; 1: weak; 2: moderate; 3 and 4: strong) and proportion of cells stained (0: 0%; 1: > 0% to ≤ 25%; 2: > 25% to ≤ 50%; 3: > 50% to ≤ 75%; 4: > 75–100%).

### Immunofluorescence Staining

For immunofluorescence staining, the same steps were followed as described above for the staining of the immunohistochemical samples, with the exception that, following incubation with the primary antibodies, the relevant Invitrogen® fluorescent secondary antibodies (Thermo Fisher Scientific, Inc., Waltham, MA, USA; cat. no. A-11034,1:400 dilution) were incubated for 2 h at 37°C. After washing with 0.1 M PBS, the slides were mounted using antifading medium (with DAPI). Slides were then checked via confocal microscopy. Confocal images were acquired using a laser scanning confocal microscope (Leica TCS SP5, Leica Microsystems GmbH, Mannheim, Germany) equipped with 561 and 633 nm lasers for excitation and a 63x oil immersion objective (1.4 NA, Leica). The IF assay was used to examine the expression and localization of TNF-α, IL-6, IFN-γ and IL-10 in ES from the sac specimens of patients with MD.

### Statistical Analyses

Data are presented as the mean values ± standard deviation. For statistical analysis, a paired Student's t-test was used for two-group comparisons, whereas Pearson's test was adopted for evaluating the relationship. *P* < 0.05 was considered to indicate a statistically significant value. For cytokine measurements, when variances among the serum groups were equal, one-way ANOVA was used to compare the groups. All data were statistically treated with SPSS version, 26.0 (IBM Corp., Armonk, NY, USA).

## Results

### Expression Levels of the Cytokines in the Various Samples

Table 1 shows the levels of IFN-γ, IL-12p70, IL-6, IL-10, TNF-α and IL-17A in the luminal fluid of the ES from the MD and the AN groups, and of the serum from the MD and AN groups and the healthy control group, as detected using a Simoa Cytokine 6-Plex Panel 1 Advantage kit. Data (pg/ml) are presented as the mean values ± SD. All cytokines were detected in samples of the diluted luminal fluid of the ES, including the MD and AN groups, and the serum from the MD, AN and healthy groups. Samples with readings below the minimum detection limit or above the maximum detection limit were assigned values of 0 pg/ml for the minimum value, or 1,000 pg/ml for the maximum value. In samples of the diluted luminal fluid of the ES, no samples were identified that were above the maximum value in either the MD group or the AN group, whereas two samples from the AN group for IFN-γ, one sample from the MD group and two samples from the AN group for TNF-α, one sample from the MD group and two samples from the AN group for IL-17A, and one sample from the MD group and three samples from the AN group for IL-12p70, exhibited values below the detection limit. In the serum samples, no samples with readings below the minimum detection limit or above the maximum detection limit were identified in any of the three groups.

**Table 1:** Cytokine levels in ES luminal fluid from MD group and AN group, and the serum from MD group, AN group and healthy control group (mean ± SD).

	**IL-6**	**IFN-γ**	**TNF-α**	**IL-10**	**IL-12p70**	**IL-17A**
MD ELF (*n* = 20)	1.371 ± 0.5427	0.2787 ± 0.0942	0.2656 ± 0.0797	0.1346 ± 0.0296	0.0870 ± 0.0265	0.0721 ± 0.0300
AN ELF (*n* = 20)	0.8939 ± 0.5290	0.1601 ± 0.0610	0.2094 ± 0.0834	0.1551 ± 0.0491	0.0773 ± 0.0367	0.0783 ± 0.0545
AN ELF (*n* = 20)	2.3370 ± 0.8382	1.4361 ± 0.3770	3.291 ± 0.6611	1.5989 ± 0.5002	0.2599 ± 0.1332	0.1528 ± 0.1886
AN serum (*n* = 20)	2.2251 ± 0.8881	1.5293 ± 0.3879	3.4988 ± 0.5981	1.7497 ± 0.4699	0.2077 ± 0.0938	0.1196 ± 0.0679
Control serum (*n* = 20)	2.1033 ± 0.7608	1.5933 ± 0.3208	3.4927 ± 0.7303	1.7255 ± 0.5332	0.2473 ± 0.1136	0.1095 ± 0.0422

*MD, Meniere's disease; AN, acoustic neuroma; ELF, ES luminal fluid; SD, standard deviation; n, number*.

**Figure 3:**
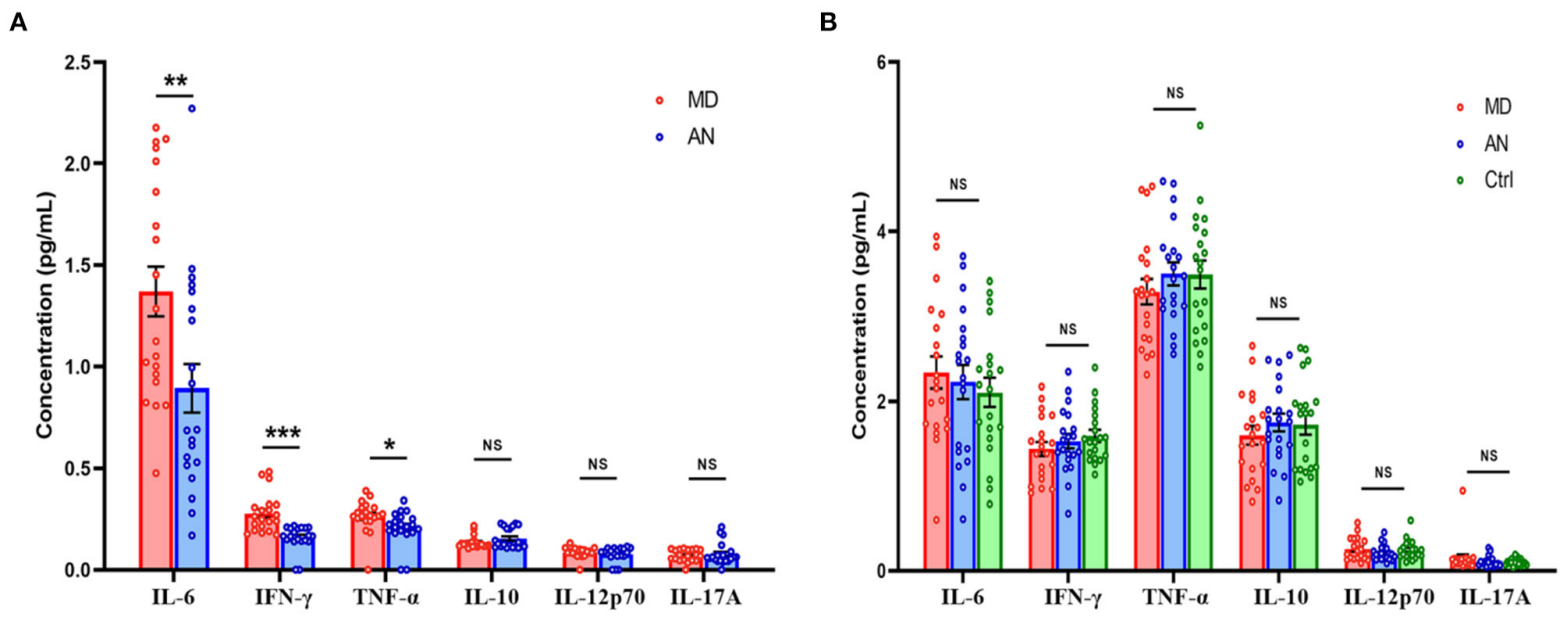
**(A)** Cytokine levels of ELF in the MD group compared with those in the AN group. **(B)** Cytokine levels of the serum in the MD group compared with those in the AN group and the healthy control group. *P < 0.05; **P < 0.01; ***P < 0.001; ES, endolymphatic sac; ELF, ES luminal fluid; MD, Meniere's disease; AN, acoustic neuroma.

Significant differences were noted in the expression levels of IFN-γ (*P* < 0.001), IL-6 (*P* = 0.008) and TNF-α (*P* = 0.036) in the luminal fluid of the ES compared between the MD and AN groups, whereas no statistically significant differences were observed in the expression levels of IL-10 (*P* = 0.154), IL-12p70 (*P* = 0.344) and IL-17A (*P* = 0.659) in the luminal fluid of the ES comparing between the MD and AN groups (Figure 3A).

Cytokine levels of serum in the MD group were then compared with those of the AN group and the heathy control group. No statistically significant differences in the expression levels of IFN-γ (*P* = 0.445), IL-10 (*P* = 0.332), IL-12p70 (*P* = 0.160), IL-17A (*P* = 0.464), IL-6 (*P* = 0.684) or TNF-α (*P* = 0.304) were identified in the serum comparing between the MD and AN groups; neither were there any statistical significant differences found in the expression levels of IFN-γ (*P* = 0.164), IL-10 (*P* = 0.444), IL-12p70 (*P* = 0.749), IL-17A (*P* = 0.323), IL-6 (*P* = 0.362) or TNF-α (*P* = 0.365) in the serum comparing between the MD group and the healthy control group (Figure 3B).

No significant correlations were observed in the expression levels of IFN-γ (r^2^ = 0.038, *P* = 0.408), IL-10 (r^2^ = 0.038, *P* = 0.409), IL-12p70 (r^2^ = 0.041, *P* = 0.394), IL-17A (r^2^ = 0.029, *P* = 0.475), IL-6 (r^2^ = 0.004, *P* = 0.798) and TNF-α (r^2^ = 0.059, *P* = 0.302) compared between the ELF and serum in the MD group. Similarly, no significant correlations in the expression levels of IFN-γ (r^2^ = 0.026, *P* = 0.499), IL-10 (r^2^ = 0.016, *P* = 0.595), IL-12p70 (r^2^ = 0.004, *P* = 0.401), IL-17A (r^2^ = 0.024, *P* = 0.512), IL-6 (r^2^ = 0.113, *P* = 0.147) and TNF-α (r^2^ = 0.025, *P* = 0.502) were observed comparing between the ELF and serum in the AN group.

### Immunohistochemical and Immunofluorescent Detection of the Cytokines in the Surgically Removed Human ES of Patients With MD and AN

**Figure 4:**
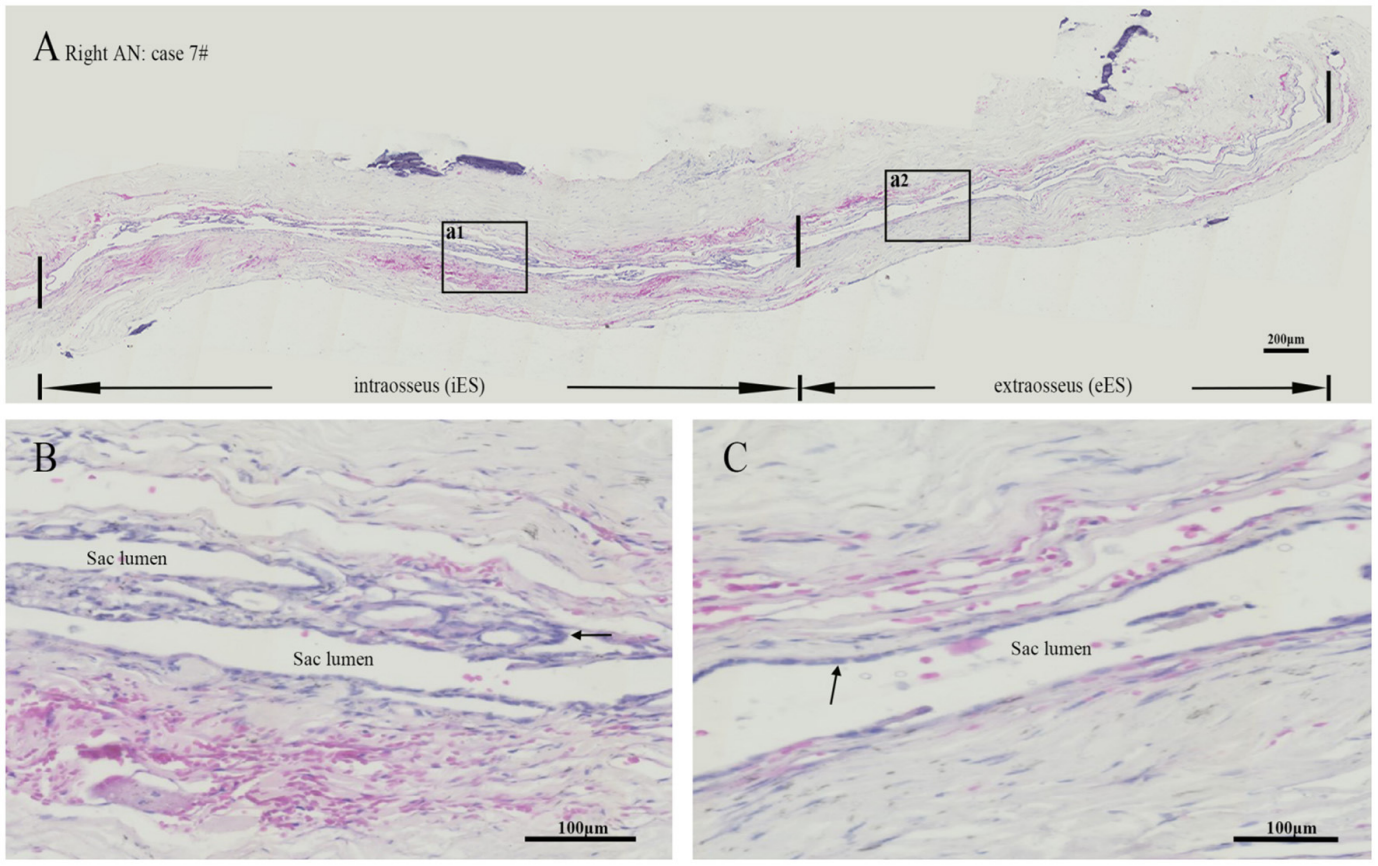
Hematoxylin-Eosin staining revealed a lumen was patent or slit-like in the intraosseous and extraosseous portions of the ES from patient no. 7 with acoustic neuroma **(A)**, whereas the framed area of a1 in **(A)** magnified to show an intact folded epithelial (arrow) lining in the lumen of intraosseous part of ES **(B)** and the framed area of a2 in **(A)** magnified to show a simple cuboidal epithelium (arrow) lining in the lumen of extraosseous portion of the ES **(C)**. ES, endolymphatic sac. Scale bars: **(A)** 200 μm; **(B,C)** 100 μm.

**Figure 5:**
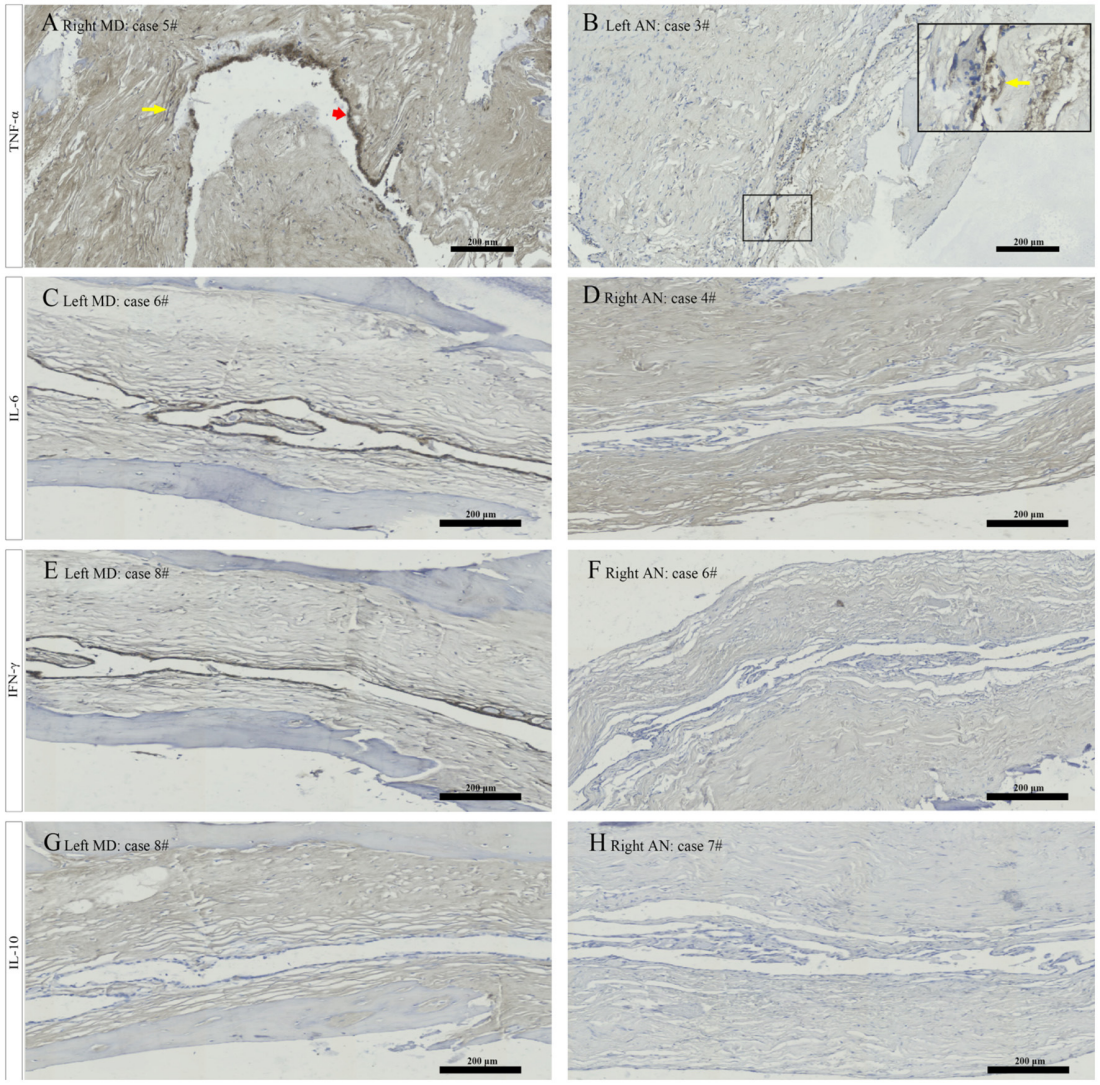
Immunostaining for TNF α showed strong staining of epithelial cells (red arrowhead) and weak staining of the subepithelial fibroblasts (yellow arrow) in patient no. 5 with right MD **(A)**, and weak staining of epithelial cells in the magnified framed area (yellow arrow) in patient no. 3 with left AN **(B)**. Immunostaining for IL 6 revealed strong signal in the ES epithelium in patient no. 6 with left MD **(C)** and no positive expression of epithelial cells in patient no. 4 with right AN **(D)**. Immunostaining from ES specimen in patient no. 8 with left MD showed strong labeling along the epithelial lining of ES for IFN γ **(E)** and a negative expression in the epithelial cells of ES for IL-10 **(G)**. Whereas a negative expression of the ES epithelium for IFN γ in patient no. 6 with right AN **(F)** and IL-10 in patient no. 7 with right AN **(H)** were detected. MD, Meniere's diseae; AN, acoustic neuroma; ES, endolymphatic sac. Scale bars: **(A–H)** 200 μm.

Morphology in all specimens was well preserved at the light microscopic level. The ES specimens in patients with MD only comprised the intermediate portion of the ES, including the distal intraosseous and proximal extraosseous (lateral side) portions of the ES tissue, which is also termed “rugose” since its epithelium is folded and contains secretory-like epithelial tubules, whereas the majority of ES specimens in patients with AN contained a substantial intraosseous and extraosseous portion of the ES (Figures 4A–C). Concerning TNF-α, positive immunostaining occurred in the epithelium of the ES, as well as in the subepithelial connective-tissue fibroblasts, showing a positive expression in 9 out of 9 ES specimens from patients with MD, including the strong staining of epithelial cells in 5 specimens (Figure 5A), moderate staining of epithelial cells in 3 specimens and weak staining of epithelial cells in 1 specimen, whereas a weak staining of epithelial cells of TNF-α (Figure 5B) in 4, and a negative expression of TNF-α in 6 ES specimens from patients with AN were detected. Concerning IL-6, this cytokine exhibited prominent staining along the epithelial lining of the sac, demonstrating a positive expression in 7 out of 9 ES specimens from patients with MD, including epithelial cells immunolabeled strongly (Figure 5C) in 4, moderately in 2 and weakly in 1, whereas a negative expression of IL-6 in all ES specimens of patients with AN was observed (Figure 5D). Regarding IFN-γ, immunoreactivity was seen in the epithelial lining of the ES in 7 out of 9 ES specimens from patients with MD, whereas strong staining in 4 and moderate staining in 3 were shown in the epithelial cells of ES, although no positive expression of IFN-γ was observed in the epithelial lining of the ES of patients with AN (Figures 5E,F). Moreover, a negative expression of IL-10 in the epithelial cells of the ES was detected in both the MD and the AN groups (Figures 5G,H).

**Figure 6:**
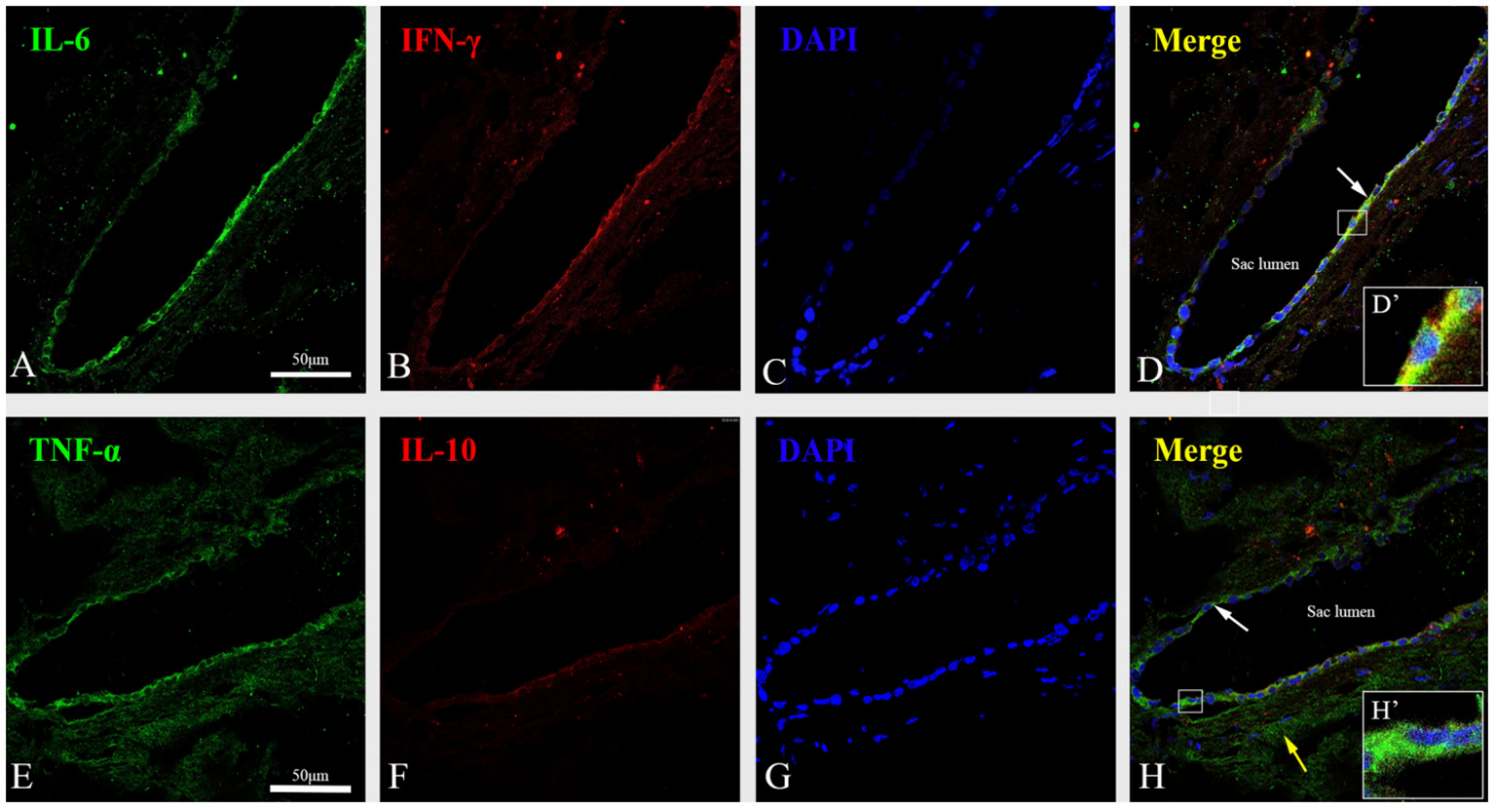
Immunofluorescent staining showed a strong cytoplasmic immunofluorescence signal for IL-6 (green) **(A)**, IFN-γ (red) **(B)** and TNF-α (green) **(E)**, and a minimal cytoplasmic immunofluorescence signal for IL-10 (red) **(F)** along with the marker of nuclei, DAPI (blue) **(C,G)** in the proximal extraosseous part of the ES in patient no. 8 with MD. **(D)** The co-localization regions of IL-6 and IFN-γ showed color in yellow in ES epithelia cells (arrow) in the merged image. The framed area is magnified in (D′), showing that IFN γ and IL-6 were mainly expressed in the cytoplasm and membrane of the ES epithelium. **(H)** Merged image of immunofluorescent labeling for TNF-α and IL-10 showed color in green in ES epithelia cells (white arrow) and the sub-epithelial tissue and Sub-epithelial fibrocytes (yellow arrow). The framed area is magnified in (H′), showing the expression of TNF-α was localized in the cytoplasm and membrane of the ES epithelium. MD, Meniere's disease; ES, endolymphatic sac. Scale bars: **(A–H)** 50 μm.

Immunofluorescent staining showed a strong cytoplasmic immunofluorescence signal for IL-6 (Figure 6A), IFN-γ (Figure 6B) and TNF-α (Figure 6E) and a minimal cytoplasmic immunofluorescence signal for IL-10 (Figure 6F) along with the marker of nuclei, DAPI (blue) (Figures 6C,G) in the epithelial cells of the ES from the sac specimens of patients with MD. Meanwhile, confocal images demonstrated that IFN-γ and IL-6 were mainly expressed in the cytoplasm and membranes of the ES epithelium (Figure 6D), whereas TNF-α immunolabeling was localized not only in the cytoplasm and membranes of the ES epithelium, but also in the sub-epithelial tissue and sub-epithelial fibrocytes (Figure 6H).

## Discussion

To the best of the authors' knowledge, this study has provided the first direct measurements of cytokines in human luminal fluid of ES. The approach of using a Simoa Cytokine 6-Plex analysis of the diluted luminal fluid of the ES was shown to be feasible, and this has provided more refined insights into the microenvironment of the ES in MD where these methods have not been used previously. The present study has highlighted the up-regulated expression of TNF-α, IL-6 and IFN-γ in the luminal fluid of ES in patients with MD through investigating the expression level of the cytokines in the ELF in patients with MD and controls. Moreover, a positive expression of TNF-α, IL-6 and IFN-γ in the epithelial cells lining the sac was detected in the majority of ES specimens from patients with MD through immunohistochemical and immunofluorescent investigation, and this confirmed the up-regulation of expression of these cytokines in the luminal fluid of ES. Additionally, the fact that no significant correlation was identified in cytokine expression between the luminal fluid of the ES and the serum suggested that specialized mechanism(s) exist that regulate the expression of cytokines in the ES. The results of the present study should contribute toward the development of novel strategies for the diagnosis and treatment of MD.

TNF-α and IL-6 are pro-inflammatory cytokines that have key functions in diverse cellular processes, including regulation of the pro-inflammatory responses and maintenance of cellular homeostasis. Previous studies have shown that TNF-α is a harmful factor for cochlea inflammation (7), and IL-6 is necessary both for B-cell development into plasma cells and their antibody production, and this is associated with infections or other inflammatory cascades (7, 29). Several studies have reported that the *in vivo* production of TNF-α, IL-1β and IL-6 has the potential to induce secondary inflammatory responses, including leukocyte infiltration, scar formation or gliosis in injured cochlea (30, 31). Moreover, elevation of the levels of TNF-α and IL-6 are considered to be involved in immune cell proliferation, prolongation of the inflammatory response and tissue remodeling in numerous middle and inner ear maladies (19, 30, 32). In the present study, the elevated levels of TNF-α and IL-6 in the luminal fluid of the ES, and a positive expression of TNF-α and IL-6 in the epithelial cells lining the sac from patients with MD compared with those of the controls, may represent a prolonged immune response and excessive inflammation with epithelial immunological injury and remodeling in the ES, where the epithelial cells lining parts of the endolymphatic spaces are crucially important for Na^+^ transport capacity (33). The ensuing cellular degeneration and dysfunction of the ES may lead to an ionic imbalance in the endolymph, resulting in the accumulation of endolymph at the cochlear duct. Recently, degenerative and hypoplastic changes in the ES of patients with MD have been directly linked to the pathogenesis of EH (34). Interestingly, altered TNF-α expression has been proposed to have a significant role in MD, and TNF-α inhibitors are being preliminarily investigated as a possibility for therapeutic intervention (35). In mouse models, systemic injection of the TNF blocker, etanercept, resulted in a reduction in the cytokine expression levels of a number of cells in the ES lumen (7) and protection against TNF-α-induced sensorineural hearing loss (SNHL) (36), whereas the loss of IL-6 production in knock-out mice was shown to suppress macrophage recruitment and decrease local inflammation (37). In another study, the blockage of IL-6 by specific humanized neutralizing antibodies was clinically used for patients with rheumatoid arthritis or inflammatory bowel disease with promising effects (38). Although the findings of the present study have provided direct evidence for the inflammatory state of the ES, more research is needed for further development of therapeutic strategies for treatment of MD.

IFN-γ is a T-cell cytokine that induces macrophages to produce a variety of inflammatory mediators and reactive oxygen and nitrogen intermediates. Several studies using cultured human ES epithelium have demonstrated changes in Na^+^ transport during the application of IL-1β (39, 40). Son et al. (41) reported that the Na^+^ transport activity mediated via the epithelial Na^+^ channel, Na^+^-H^+^ exchanger, was significantly decreased following the application of IFN-γ. On the basis of these experimental studies, an up-regulated expression of IFN-γ in the luminal fluid of ES from patients with MD compared with those of controls, and a positive expression of IFN-γ in the epithelial cells lining the sac, could be expected to decrease Na^+^ transport in the ES lumen, which may consequently cause higher luminal [Na^+^] and an increased luminal fluid volume, since water shift usually accompanies Na^+^ movement. Recently, loss or absence of aldosterone regulated Na^+^ transport proteins in degenerative and hypoplastic ES pathology has been exhibited in the ES specimens of patients with MD (34). This may support one of the pathological mechanisms for EH under certain inflammatory conditions of the ES. Accumulating evidence supports the link between the pro-inflammatory cytokine profile in peripheral blood mononuclear cells and the pathogenesis of MD, including higher basal levels of IL-1β, IL-6 and TNF-α and a release of TNF-α induced by both *Aspergillus and Penicillium* extracts in several patients with uni- or bilateral MD (35). The allelic variation in rs4947296, at 6p21.33, developing a nuclear factor-κB (NF-κB)-mediated inflammatory response, are supposed to be related to an abnormal inflammatory response at the ES in bilateral MD (21), and there are higher levels of cytokines/chemokines in patients with MD with high basal levels of IL-1β (42). Additionally, significant differences in CD4, CD4/CD8 and CD23 lymphocyte subpopulations and IFN-γ and IL-4 levels in the peripheral blood of patients with MD were observed when they were compared with controls (43). However, the present study showed no statistical differences in IFN-γ, IL-10, IL-12p70, IL-17A, IL-6 and TNF-α levels in the serum comparing among the MD and AN groups and a healthy control group, indicating an aberrant systemic pro-inflammatory response was not found in these unilateral patients with MD. Moreover, no correlation was found between the expression of cytokines in the luminal fluid of the ES and that in the serum from patients with MD and patients with AN, suggesting the existence of different mechanisms involving the modulation of the expression of cytokines in the ES and blood. Considering that increases in IFN-γ, TNF-α and IL-6 levels were observed in the luminal fluid of ES in these patients with MD, this could indicate that a specialized mechanism independent of the systemic cytokine response may be causing the abnormal cytokine-mediated inflammatory response at the ES, demonstrating increased levels of IFN-γ, TNF-α and IL-6 in the ES lumen and a positive expression of TNF-α, IL-6 and IFN-γ in the epithelial cells lining the sac. On the other hand, this increased production of cytokines could also affect or represent immune activity that is occurring in ES. Several studies have shown the ES could be a target organ attacked by the allergic reaction (5), viral or bacterial infection (9–11), autoimmunity (13–15), otitis media-induced inflammatory products and toxins (44), circulating immune complexes (45), genetic predisposition to altered NF-κB-mediated inflammatory responses (21) and distinct and altered cytokine profiles (16), resulting in the damage of the epithelial layers surrounding the ES space and the dysfunction of ES. However, it was noticed that no statistically significant difference in the expression level of IL-10 in the ELF between the MD and AN group and a negative expression of IL-10 in the epithelial cells of the ES in patients with MD were observed. It is known that IL-10 is a potent anti-inflammatory cytokine. Induction of IL-10 often occurs together with pro-inflammatory cytokines, although pathways that induce IL-10 may actually negatively regulate these pro-inflammatory cytokines (46). The reason for an unaltered expression level of IL-10 in the ES of patients with MD in whom an up regulated expression of TNF-α, IL-6 and IFN-γ in the ES was detected is unclear, which need to be studied further.

The treatment of MD using EDB has been shown to be effective for the control of symptoms of MD without any noticeable cochlear and vestibular damage (47, 48), thereby providing opportunities for sampling ELF and ES specimens in order to investigate the protein composition and molecular biomarkers in human ES of MD. However, superior levels of medical expertise and carefulness are required in sampling the luminal fluid of the ES from human subjects such that contaminating the samples with surrounding tissue fluids and blood is avoided. It must be acknowledged that the samples could have been contaminated from surrounding tissues, fluids and blood. As we have mentioned above, one sample in patient with MD and two samples in patients with AN had to be abandoned due to the suspicion of blood contamination. Recently, Ölander et al. (49) reported an *in vivo* and *in situ* sampling probe technique for collecting ES endolymph samples, which provided a novel way for sampling of proteins in the luminal fluid of the ES. To reduce the risk of contamination of samples, they proposed that the area for sampling was first thoroughly rinsed with Ringer's acetate solution before final drilling and opening of the vestibular aqueduct, and the sampling probe was covered with a metal sleeve that was removed as the probe was inserted into the ES. However, in contrast with the procedure of obtaining 200 μl sample (diluted luminal fluid) from 200 μl normal saline infused into the ES that has been reported in the literature (14, 15), 2 μl sample obtained from 2 μl sterile water infused into the ES in the present study could possibly reduce the risk of the contamination of samples with other body fluids. Moreover, a standard volume (2 μl) of ELF sampled from each person recruited in our study also had an advantage in terms of quantitative analysis of the amounts of protein in the controls and patients. Recently, Warnecke et al. (50) reported that multiplex protein analyses were feasible in very small samples (~1 μl or less) of human perilymph fluid and was able to identify marker proteins of sterile inflammation as well as of the innate and adaptive immune system using Luminex-based multiplex arrays (human 27-Plex). In the present study, the use of a single-molecular enzyme-linked immunosorbent assay, which has been reported to have the potential to detect soluble immune signaling molecules at an ultralow detection limit of 0.67 fM (0.012 pg/ml) (51, 52), enabled us to perform the quantitative detection of subfemtomolar concentrations of cytokines in 2 μl diluted luminal fluid of ES, thereby demonstrating the feasibility of identifying biomarkers of inner ear disease in very small amounts of diluted luminal fluid of ES using this highly sensitive and specific technique.

There were, however, certain limitations associated with the present study. This is an ambitious experimental setup due to: (i) the difficulties of identification of the lumen in ES, and (ii) the skill needed to take samples of the diluted luminal fluid without any intraoperative contamination occurring. Thus, we need to consider that the area surrounding the sac remains almost dry during luminal fluid sampling, as shown in Figures 2A,B. Through using bone wax, electrocautery and careful suction for cleaning, the risk of intra-surgical contamination can be reduced. Theoretically, the use of the luminal fluid of ES from patients with normal hearing as a control would have improved our study; however, sampling the luminal fluid of ES from patients with normal function of the inner ear would have been impossible for ethical reasons. In addition, the interpretation of the results in this study was possibly affected by using the ELF from patients with AN as a control for a comparative analysis of the cytokine levels in the ELF in patients with MD, since an up-regulation of intracochlear levels of TNF-α secreted by tumors possibly present in patients with AN are presumed to result in AN-associated SNHL (53).

In conclusion, the up-regulated expression of TNF-α, IL-6 and IFN-γ in the luminal fluid of ES, in the absence of any aberrant systemic pro-inflammatory response, has provided direct evidence for an increased local inflammatory response at the ES in these patients with unilateral MD, and this phenomenon was validated by demonstrating a positive expression of TNF-α, IL-6 and IFN-γ in the epithelial cells lining the sac in human ES specimens from patients with MD using immunohistochemical and immunofluorescent analysis. However, no significant correlation was identified between the cytokine levels of the ELF and the cytokine levels of the serum, suggesting that a specialized mechanism for the regulation of cytokines is likely to be operating in the ES.

## Data Availability Statement

The raw data supporting the conclusions of this article will be made available by the authors, without undue reservation.

## Ethics Statement

The studies involving human participants were reviewed and approved by Medical Ethics Committee of the Second Xiangya Hospital. The patients/participants provided their written informed consent to participate in this study. Written informed consent was obtained from the individual(s) for the publication of any potentially identifiable images or data included in this article.

## Author Contributions

CH and ZZ designed and accomplished the experiments together. QW provided technical advice. XP and WLi conducted the experiments. WLiu participated in the revision of the manuscript. AP being the Principal Investigator of the research project, directed the design, and the procedure of the experiments. WJ and LH analyzed data and drafted the paper. All authors approved the final version of the manuscript.

## Funding

The authors were funded by National Natural Science Foundation of China (Grant No. 81570928).

## Conflict of Interest

The authors declare that the research was conducted in the absence of any commercial or financial relationships that could be construed as a potential conflict of interest.

## Publisher's Note

All claims expressed in this article are solely those of the authors and do not necessarily represent those of their affiliated organizations, or those of the publisher, the editors and the reviewers. Any product that may be evaluated in this article, or claim that may be made by its manufacturer, is not guaranteed or endorsed by the publisher.

## Abbreviations

MD: Meniere's disease
EH: endolymphatic hydrops
ES: endolymphatic sac
ELF: ES luminal fluid
AN: acoustic neuroma
EDB: endolymphatic duct blockage
TNF-α: tumor necrosis factor α
IFN-γ: interferon-γ
IL: interleukin
Simoa: single-molecular array
RGP: resorufin β-D-galactopyranoside
PBST: triton-phosphate buffered saline
DAPI, 4′: 6-diamidino-2-phenylindole
ANOVA: analysis of variance
IF: immunofluorescence
SNHL: sensorineural hearing loss.
